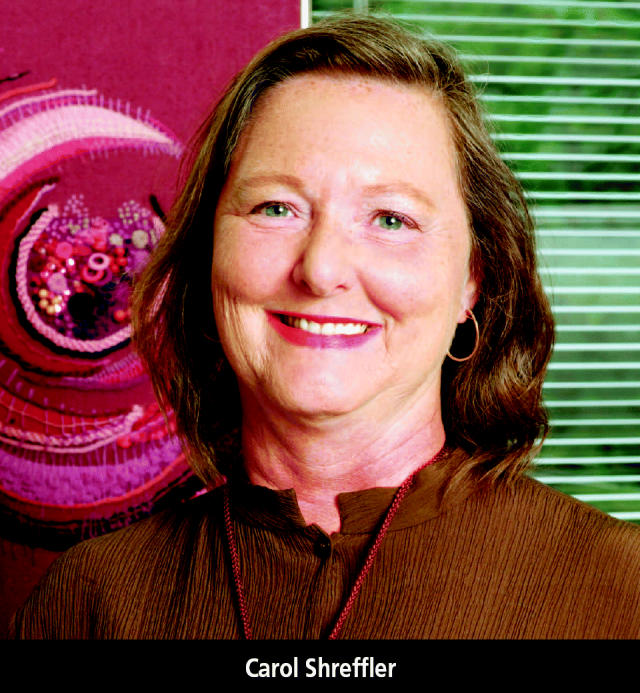# NIEHS Dual-Degree Predoctoral Fellowships for Training Clinician–Scientists

**Published:** 2004-08

**Authors:** Carol Shreffler

**Affiliations:** **PROGRAM ADMINISTRATOR** shreffl1@niehs.nih.gov Training and fellowship grants

Ruth L. Kirschstein National Research Service Awards issued under the Dual-Degree Predoctoral Fellowships for Training Clinician–Scientists program provide training for clinical scientists. First announced by the NIEHS in 1999, the program was reissued in 2001 under Program Announcement PA-01-132 (**http://grants.nih.gov/grants/guide/pa-files/PA-01-132.html**). The program offers fellowship and stipend support to trainees enrolled in a combined MD/PhD or MD/MPH program at an accredited medical school. Trainees must be conducting basic or clinical research in environmental medicine or on a specific environmentally related disease or disorder.

For this fellowship, environmental medicine is the area of medicine concerned with the development and application of knowledge directed at the etiology, diagnosis, pathophysiologic progression, treatment, and prevention of adverse effects from exposure to toxic agents. Adverse effects may include an identifiable disease, disorder, or decrement in mental or physical function. Environmental medicine is a cross-cutting issue in the medical arena in that it is problem-oriented and does not focus on a particular discipline, specialty, disease, or organ system. As the development of a disease or disorder can be viewed as resulting from the interplay of genetic and environmental factors over the life span of the individual, potent environmental influences may manifest at many stages of life: as a gamete, as an embryo, during epigenesis, *in utero*, during childhood, during adulthood, or with aging.

Linking exposures to chemical, physical, or biologically derived toxicants in the environment to clinical outcomes in humans continues to be a research focus for the NIEHS. The problems addressed are complex and of great public interest. However, the standard courses of study at most medical schools do not provide the experience to approach these issues. The NIEHS has therefore not supported a large number of physicians in its research portfolio. Individual fellowships for combined degree seekers is viewed as a way to develop highly trained clinical scientists who will be available to conduct the next generation of environmental health research, which is expected to emphasize the translation of animal- and cellular-level laboratory research to the clinical setting.

The NIEHS continues to enthusiastically support the dual-degree predoctoral fellowship program. So far 15 awards have been made to medical students at 11 medical schools. Applications are particularly sought for clinical and translational research in environmental medicine.

## Figures and Tables

**Figure:**